# ^99m^Tc-radiolabeled GE11-modified peptide for ovarian tumor targeting

**DOI:** 10.1186/s40199-017-0179-8

**Published:** 2017-05-02

**Authors:** Najmeh Rahmanian, Seyed Jalal Hosseinimehr, Ali Khalaj, Zohreh Noaparast, Seyed Mohammad Abedi, Omid Sabzevari

**Affiliations:** 1 Department of Radiopharmacy, Faculty of Pharmacy, Tehran University of Medical Sciences, Tehran, Iran; 2 Department of Radiopharmacy, Faculty of Pharmacy, Mazandaran University of Medical Sciences, Sari, Iran; 3 Department of Medicinal Chemistry, Faculty of Pharmacy, Tehran University of Medical Sciences, Tehran, Iran; 4 Department of Radiology, Faculty of Medicine, Mazandaran University of Medical Sciences, Sari, Iran; 5 Department of Toxicology, Faculty of Pharmacy, Tehran University of Medical Sciences, Tehran, Iran

**Keywords:** GE11 peptide, ^99m^Tc, Molecular imaging, HYNIC, Radiopharmaceuticals

## Abstract

**Background:**

Ovarian cancer is a serious threat for women health and the early diagnosis of this cancer might improves the survival rate of patients. The use of the targeted radiopharmaceuticals could be a non-invasive and logical method for tumor imaging. The aim of this study was to radiolabel GE11 peptide as a new specific probe for imaging of ovarian tumor.

**Methods:**

HYNIC-SSS-GE11 peptide was labeled with ^99m^Tc using tricine as a coligand. The ^99m^Tc-tricine-HYNIC-SSS-GE11 peptide was evaluated for specific cellular binding in three cell lines with different levels of EGFR expression. Tumor targeting was assessed in SKOV3 tumor bearing mice.

**Results:**

By using tricine as a coligand, labeling yield was more than 98% and the stability of the radiolabelled peptide in human serum up to 4 h was 96%. The in vitro cell uptake test showed that this radiolabeled peptide had a good affinity to SKOV3 cells with dissociation constant of 73 nM. The in vivo results showed a tumor/muscle ratio of 3.2 at 4 h following injection of ^99m^Tc-tricine-HYNIC-SSS-GE11 peptide.

**Conclusions:**

Results of this study showed that ^99m^Tc-tricine-HYNIC-SSS-GE11 peptide could be a promising tool for diagnosis and staging of ovarian cancer.

**Graphical Abstract:**

^99m^Tc-tricine-HYNIC-SSS-GE11, a novl targeted agent for ovarian tumor imaging
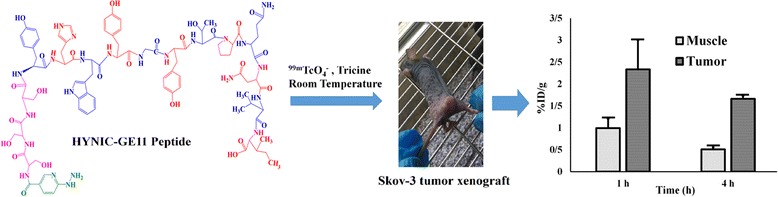

## Background

Ovarian cancer has been reported as the fifth major cause of cancer related death and the eight most common cancer among women. Since the symptoms of the ovarian cancer in early stages are not clear, nearly 50% of patients are diagnosed in advanced stages (stage III/IV) whereas patient are over 65 years old [1, 2]. While early diagnosis may increase survival times of 90% of patients to 5 years, late diagnosis at stage 3 increases survival times of only 33% of patients to 5 years. Considering that ovarian cancer is frequently diagnosed at stage III or IV, a convenient screening method for early detection is highly desirable [3]. Recent approaches have focused attention to tumor targeting agents for identification of tumor cells. It has been shown that in approximately 70% of ovarian cancers there is an overexpression of Epidermal Growth Factor Receptors (EGFRs) which belongs to receptor kinase family and consist of 4 members, namely: HER1 (Human Epidermal Growth Factor Receptor 1; EGFR), HER2/neu, HER3 and HER4 [4]. Binding of EGF to EGFR is followed by homo or heterodimerization of the receptors which activate intracellular cascades and as a consequence result in cellular responses such as proliferation, differentiation and survival [5]. Overexpression of EGFR family which has been found in many human malignancies such as gastric, Non-Small Cell Lung Cancer (NSCLC), Squamous Cell Carcinoma of the Head and Neck (SCCHN), breast, ovarian, prostate, colorectal, esophageal, bladder and renal cancers result in tumor recurrence which decreases patient survival [6, 7]. Currently monoclonal Antibodies (mAbs) such as trastuzumab, pertuzumab and cetuximab are being used for treatment of tumors associated with overexpression of EGFR [8]. Measurement of EGFR levels have been described as a reliable method to follow up patient responses and to evaluate treatment efficacy following therapy with EGFR inhibitors. The radionuclide molecular imaging as a non-invasive method has been used to detect EGFR expression [9]. Radiolabeled peptides, proteins and antibodies such as [^111^In] Bz-DTPA-hEGF, ^99m^Tc-EGF, ^64^Cu-DOTA-cetuximab, ^111^In-DTPA-CHX-A”-cetuximab, ^111^In-MAb 225, ^99m^Tc-8B6 nanobody, and ^111^In-DOTA-Affibody molecule are some examples of EGFR specific radionuclide imaging agents. While ^99m^Tc-radiolabeled antibodies have advantages of using readily available radionuclide through a generator at low cost which emits only gamma radiation of low energy of 140 Kev, they have some limitations such as slow tumor accumulation, slow blood clearance rate and high abdominal accumulation and as a result diminished target-non target contrast. Small molecular size peptides which have low side effects and good clearance pattern from blood are good targeting and imaging agents [10]. In 2005 Li et al. reported a novel dodecapeptide YHWYGYTPQNVI (GE11) as an EGFR targeting ligand which binds effectively to epithermal growth factor receptor without activating EGFR-mediated signaling pathway [11]. Selectivity and sensitivity of GE11 for EGFR makes it as a promising vector for systematic delivery of therapeutic pharmaceuticals [12–15] and also for specific delivery of imaging agents such as radioactive tracers including ^18^F, ^64^Cu and ^131^I [16–18]. Since high resolution for tumor visualization depends on high radioactivity uptake in the tumor and low uptakes in non-target tissues such as liver which might also blind metastatic lesions [19] several studies have indicated that pharmacokinetic modification of the imaging contrast through the use of an appropriate linker result in increased tumor uptake, reduced non-target tissues uptake and better tumor visualization [20, 21]. Among different linkers the use of Seryl-Seryl-Serine (SSS) sequence as spacer has shown remarkable effects on biodistribution by reducing liver accumulation and rapid clearance of the radiolabelled peptides [20, 21]. In this study SSS-GE11 was conjugated with hydrazinenicotinamide (HYNIC) and labeled with ^99m^Tc using tricine as a coligand. The in vitro specificity of the radiolabelled peptide for binding to EGFR was determined in SKOV3, A549, and MCF-7 cell lines, of which SKOV3 ovarian carcinoma cells accumulated higher amount of the radiolabeled peptide and was used for in vivo tumor targeting in nude mice. In addition, biodistribution of the radiolabeled peptide was investigated in both normal and nude mice in order to determine the effects of Seryl-Seryl-Serine (SSS) sequence as spacer on liver accumulation and renal clearance of the radiolabeled peptide.

## Methods

### Instrumentation and materials

The HYNIC-SSS-GE11 was purchased from ProteoGenix (Schiltigheim, France). ^99m^Tc was obtained from a ^99^Mo/^99m^Tc radionuclide generator (Pars Isotope, Iran). Ammonium acetate, sodium citrate, acetonitrile (HPLC grade), and Methyl Ethyl Ketone (MEK) were obtained from Merck company (Darmstadt, Germany). Trifluoroacetic acid (TFA), anhydrous tin (II)-chloride and tricine were from Sigma-Aldrich company (St. Louis, MO, USA). Double distilled deionized water were used for the preparation of aqueous solutions. Lablogic mini scan TLC scanner (Sheffield, UK) was used for quantification of the distribution of radioactivity which was determined by Instant Thin Layer Chromatography on Silica Gel (ITLC-SG) strips and the resulting data were analyzed by Laura image analysis software. Radioactivity in the sample was measured using a gamma counter with a NaI(Tl) gamma detector (Delshid, Tehran, Iran). Knauer HPLC system (Berlin, Germany) was used for analytical reversed-phase high-performance liquid chromatography (RP-HPLC) and the HPLC analyses of the radiolabeled peptides were performed on a Lablogic radioactivity gamma detector with a Eurospher 100–5 C18, 4.6 × 250 mm (Knauer, Berlin, Germany) column. Elution of RP-HPLC was performed with a solvent system consisting of: 0.1% TFA in acetonitrile (solvent A) and 0.1% TFA in water (solvent B).

Human ovarian cancer (SKOV3), Non–Small Cell Lung Cancer (NSCLC) (A549) and human breast cancer cell lines (MCF-7) were obtained from the Pasture Institute of Iran and Iranian Genetic and Biological Resource Center and cultured at 37 °C in the presence of 5% CO_2_ in Dulbecco’s Modified Eagle’s Medium (DMEM) (Gibco, Paisley, UK) supplemented with 10% Fetal Bovine Serum (FBS) and 100 μg/mL penicillin–streptomycin (Gibco, UK). HER2-specific antibody (trastuzumab), EGFR-specific antibody (cetuximab), and a CD20-specific antibody (rituximab) were from Roche (Switzerland).

All animal studies were carried out in accordance with the national animal protection regulation and approved by the Research Committee of Tehran University of Medical Sciences with approval code of 9112080151–131701.

### Radiolabeling of HYNIC-SSS-GE11 conjugate with ^99m^Tc

Radiolabeling of the peptide was performed according to the reported procedure with some modifications [22]. Briefly, 10 μg of HYNIC-SSS-GE11 was dissolved in 100 μL of 0.5 M ammonium acetate buffer of pH 6. Solution of tricine (10 mg in 0.5 M ammonium acetate buffer of pH 6) as a coligand for HYNIC was added to the HYNIC-SSS-GE11 and mixed with 100–1480 MBq of fresh ^99m^Tc-pertecnetate solution and 40 μg of SnCl_2_. 2H_2_O (1 mg/mL in 0.1 N HCl). The mixture was incubated at room temperature for 30 min.

### Quality control assessment

The labeling yield and radiochemical purity were assessed by analytical HPLC and Instant Thin Layer Chromatography on Silica Gel (ITLC-SG). For analytical HPLC a gradient elution of 0.1% TFA in acetonitrile (A) and 0.1% TFA in water (B) was employed as follows: 0–10 min: (10–25% A and 90–75% B); 10–15 min: (25–50% A and 75–50% B); 15–20 min: (50–90% A and 50–10% B) for a total time of 20 min and flow rate of 1.0 mL min^−1^. All solvents were filtered and degassed prior to passage through the column. ITLC-SG was run alongside by the use of different mobile phases such as methyl ethyl keton for free pertechnetate (*R*_f_ = 1), acetonitrile 50% for reduced hydrolyzed technetium (*R*_f_ = 0) and 0.1 M sodium citrate buffer of pH 5.5 for free ^99m^Tc-coligand (*R*_f_ = 1) [23].

### Stability assessment

The stability of ^99m^Tc-tricine-HYNIC-SSS-GE11 peptide in solution at different time intervals (shelf life) was evaluated by dilution of the reaction mixture up to 1 ml with ammonium acetate buffer of pH 6 and incubation at ambient temperatures up to 24 h. The radiochemical purity of the diluted reaction mixture was analyzed by ITLC. All experiments were carried out in triplicates.

Serum stability of the peptide was also determined following addition of 100 μl of fresh human serum to 20 μl of the reaction mixture and incubation for 1 and 4 h at 37 °C. Plasma samples were then treated with 500 μl of a mixture of acetonitrile and ethanol (1:1), centrifuged (14,000 rpm, 6 min), filtered (0.22 μm) and degradation of the radiolabeled peptide was assessed in supernatant by RP-HPLC [24].

### Cellular specific binding

The in vitro specificity of ^99m^Tc-tricine-HYNIC-SSS-GE11 for binding to EGFR was determined by using three cell lines in SKOV3, A549, and MCF-7 according the reported method with some modifications [25]. Briefly, the cell lines were cultivated in Dulbecco’s DMEM-high glucose supplemented with 10% (v/v) FBS at 37 °C in a humidified incubator in the presence of 5% CO_2_. The cells were trypsinised using a trypsin-EDTA solution and seeded in 12 well plates with a cell density of 5 × 10^5^ cells per dish and incubated for 24 h. On the day of experiment, cells were washed with cold serum-free medium or PBS and incubated with radiolabeled peptide at 37 °C for 2 h. Incubation was interrupted by the removal of the medium which was washed twice with 1 ml of incomplete cold medium. The cells were then trypsinized and diluted to 1 mL with complete medium. The supernatant was collected and the radioactivity was measured by a gamma counter. For determination of the specific binding of ^99m^Tc-tricine-HYNIC-SSS-GE11, the SKOV3 cells were pre-incubated with 500-fold excess of the unlabeled peptide at 37 °C for 30 min prior to the addition of radiolabeled peptide. Blocking experiments were also performed in the presences of trastuzumab, cetuximab and rituximab that are HER2, EGFR and CD20 specific antibodies respectively.

### Cellular internalization

SKOV3 cells were seeded in the single well plates (1 × 10^6^ per well) and incubated with 80 nM of the radioconjugated peptide at 37 °C for 5 min and 0.5, 1, 2 and 4 h. All experiment were performed in triplicates. Incubation was interrupted by the removal of the medium which was washed twice with ice-cold incomplete medium. The cells were then treated twice with 0.5 ml of urea buffer of pH 2.5 for 5 min on the ice to remove the surface receptor-bound fraction. Then, the cells were incubated with 0.5 mL of 1 M NaOH at 37 °C for 10 min. The cell debris were collected, and the dishes were washed with 0.5 mL of NaOH solution and the radioactivity of all collected supernatant were measured by the automated gamma counter. It should be noted that the radioactivity in the urea buffer and alkaline solution were considered as membrane bound and internalized, respectively [26, 27].

### Dissociation constant

The affinity of ^99m^Tc–tricine-HYNIC–SSS-GE11 was evaluated using saturation binding assay. For this purpose the SKOV3 cells were seeded in 24-well plates (25 × 10^4^ cells/well), treated with increasing concentrations of ^99m^Tc-tricine-HYNIC–SSS-GE11 (5, 10, 30, 65, 100, 150, 225 and 300 nM) and incubated at 37 °C in the presence (for non-specific binding) or absence (for total binding) of the unlabeled peptide. Three dishes were used for total binding and one dish containing 500-fold excess of highest concentration of radiolabeled peptide was used for non-specific binding. After 60 min of incubation, the cells were rinsed with cold serum-free medium and harvested by using a trypsin–EDTA solution. The bound radioactivity was determined with a gamma well counter and the binding data were analyzed by non-linear regression using GraphPad Software Prism version 5.04 for windows (GraphPad Software Inc., California, and USA). The equilibrium dissociation constant (Kd) and the maximum binding capacity (Bmax) of the receptor were obtained by saturation analysis. .

### Biodistribution study in normal mice

Normal female NMRI mice (20–30 g, Mazandaran animal center institute, Sari, Iran) were used in this study and randomly divided into 3 groups of 4. 100 μL of the solution containing 1 μg of the radiolabeled peptide was intravenously injected to the tail vein of each mouse. Mice were euthanized through intraperitoneal injection of ketamine and xylazine at 1, 4 and 24 h after injection. Blood was collected from the heart after deep anesthesia by a syringe which had been washed with heparin. Thereafter, organ samples including; lungs, stomach, liver, kidneys, bone, muscle, salivary glands, heart, spleen and intestines were removed, weighted and the radioactivity of each sample was measured. The tissue uptake of all organs except intestine was calculated as percent of injected dose per gram tissue (% ID/g) and for the intestine was calculated as % ID of the whole sample.

### Biodistribution study in nude mice bearing SKOV3 human ovarian cancer xenografts

The in vivo tumor-targeting of the radiolabeled peptide was studied in female nude mice bearing SKOV3 cancer xenografts. For tumor induction, 10 × 10^6^ SKOV3 cells were subcutaneously injected into the right hind leg of nude mice and tumor was allowed to grow for about 4 weeks. In the day of experiment, the average tumor size was 0.63 ± 0.2 g and the mice were randomly divided into three groups of 4 mouse each. The mice were injected intravenously 1 μg of the radiolabelled peptide. At 1 h and 4 h after injection, the mice were sacrificed and the tumor and other tissues were dissected. Blocking experiment was also performed using three nude mice which were subjected to injection of the excess amount (500 μg/50 μl) of the unlabeled peptide 30 min before radioconjugate peptide injection.

## Results

### Radiolabeling of HYNIC-SSS-GE11 with ^99m^Tc

**Fig. 1:**
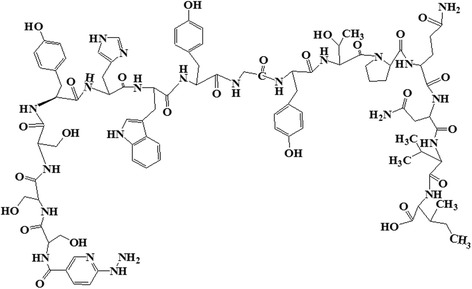
Chemical structure of HYNIC-SSS-GE11

**Fig. 2:**
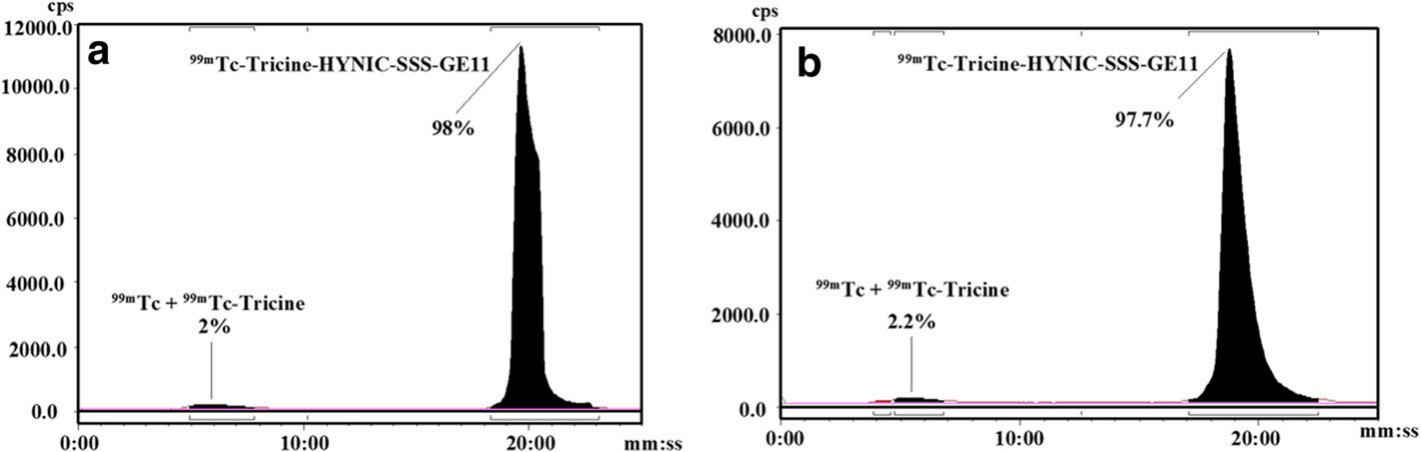
Radio-HPLC analyses of ^99m^Tc-tricine-HYNIC-SSS-GE11 in solution (**a**) and serum (**b**) 1 h post labeling

HYNIC-SSS-GE11 was labeled with ^99m^Tc using tricine as coligand and SnCl_2_ as reducing agent (Fig. 1). Various factors such as pH and type of buffer, amount of SnCl_2_, temperature and incubation time had influences on the radiochemical purity of ^99m^Tc-HYNIC-SSS-GE11. Radiochemical yield was not greater than 80% in water, normal saline, phosphate-buffered saline of pH 7 and ammonium acetate of pH 5.2 but was higher than 98% in ammonium acetate buffer of pH 6 containing tricine (10 mg), SnCl_2_ (40 μg) and ^99m^TcO_4_^−^Na (5–40 mCi). Radiochemical purity (RCP) determined after 1, 2 and 4 h by ITLC were found to be 97, 96 and 95% respectively. Also RCP assessed by radio HPLC was found to be 97 and 95% at 1 and 4 h respectively, considering 1–2% reduced hydrolyzed technetium (Fig. 2a). Experimental results showed acceptable radiochemical purity on the basis of the presence of a single radioactivity peak corresponding to ^99m^Tc-HYNIC-SSS-GE11 with a retention time of 18–20 min.

### Stability assessment in solution and serum

**Fig. 3:**
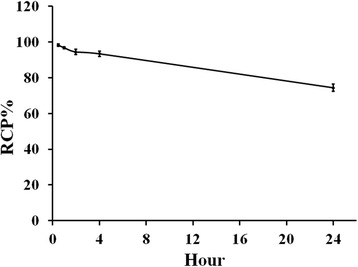
Stability of ^99m^Tc-tricine-HYNIC-SSS-GE11 in solution over time

The radiolabeled conjugate solution showed a stability of approximately 93%, within 4 h, in solution (Fig. 3). Also, the stability of radiolabeled conjugate was evaluated in human serum for 1 and 4 h by radio HPLC (Fig. 2b). ^99m^Tc-HYNIC-SSS-GE11 diluted in human serum showed stability of approximately 96% over 4 h. Based on the results, it can be concluded that the radiolabeled conjugate demonstrated acceptable stability in solution and human serum for 4 h.

### Cellular specific binding

**Fig. 4:**
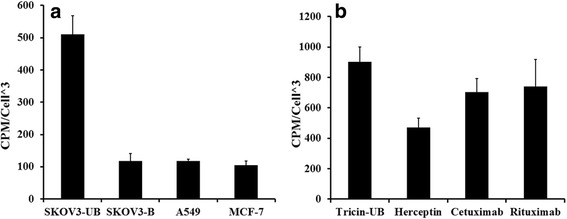
**a** Specific binding of ^99m^Tc-tricine-HYNIC-SSS-GE11 in three cell lines with different level of EGFR expression: SKOV3 (*high* HER2), A549 (*high* EGFR), MCF-7 (negative). Blocking experiment was performed in the presence of the excess (500-fold molar) of non-radiolabeled peptide. **b** Specific binding of ^99m^Tc-tricine-HYNIC-SSS-GE11 in SKOV3 cells in the presence of (500-fold molar excess) and absence of the specific anti-body; trastuzumab (anti-HER2), cetuximab (anti-EGFR) and rituximab (anti-CD20)

To confirm the specificity of the binding of radiolabeled peptide to EGFR receptor, three cell lines with different level of EGFR expression (A549: high EGFR, low HER2; SKOV3: high HER2; MCF-7: very low EGFR and HER2 expression) were used. Results showed that there was significant differences in in vitro tendency of different cells for binding to radiolabeled peptide. Based on the results, ^99m^Tc-tricine-HYNIC-SSS-GE11 had higher tendency to bind to SKOV3 than A549 which in turn had higher tendency than MCF-7 cells. Accumulation of the radiolabeled peptide in SKOV3 cell line compared to MCF-7 cell line was 5 times higher and compared to A549 cells was 4.4 times higher (Fig. 4a). On the other hand, the binding specificity tests demonstrated that the binding of ^99m^Tc-HYNIC-SSS-GE11 was receptor mediated because pre-saturation of the SKOV3 cells with 500-fold excess molar of the cold peptide decreased the specific binding to 25% (Fig. 4a).

These results also revealed that the SKOV3 cells accumulated a higher amount of radioactivity resulting from receptor binding to the radiolabeled peptide. In addition, when SKOV3 cells pre-incubated with excess molar of antibodies such as cetuximab, trastuzumab and rituximab, specific binding decreased significantly by trastuzumab as a HER2 specific antibody (Fig. 4b).

### Cellular internalization

**Fig. 5:**
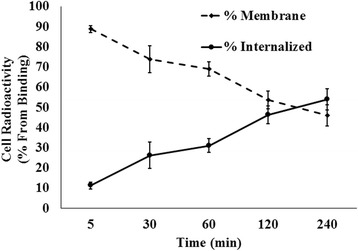
Time dependency of internalization of ^99m^Tc-tricine-HYNIC-SSS-GE11 in SKOV3 cells expressed as % of bound activity

The results of cellular internalization experiments are summarized in Fig. 5. The main source of the intracellular radioactivity was from the cell-bound radioactivity. Internalization of the radiolabeled peptide by SKOV3 cells was evaluated at 37 °C up to 4 h. Results showed that the fraction of internalized radioactivity increased with the time. Totally, about 52% of the radiolabeled peptide from membrane-bound conjugate internalized the cells up to 4 h.

### Dissociation constant

**Fig. 6:**
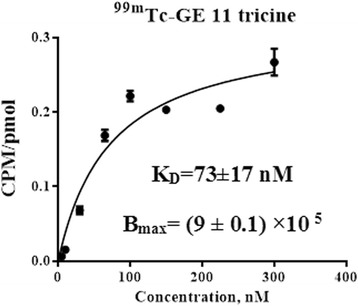
Affinity assessment of ^99m^Tc-tricine-HYNIC-SSS-GE11 in SKOV3 cell line by saturation binding analysis. Increasing concentration of ^99m^Tc-tricine-HYNIC-SSS-GE11 were added to dishes with SKOV3 cells. The cells were then harvested and bound radioactivity (*y-axis*) was determined. The errors presented in the insert are standard errors of the curve fit

The binding affinity of ^99m^Tc-tricine-HYNIC-SSS-GE11 to SKOV3 cells was determined by treating cells with increasing concentration of radiolabeled peptide. The specific binding was calculated by subtracting the non-specific binding radioactivity from total binding activity. The saturation binding curve of ^99m^Tc-tricine-HYNIC-SSS-GE11 to SKOV3 cells is shown in Fig. 6. The analysis of data revealed the radiolabeled peptide bound to receptors with a dissociation constant of 73 ± 17 nM and B_max_ of (9 ± 0.1) × 10^5^.

### Biodistribution in normal mice

The biodistribution of ^99m^Tc-tricine-HYNIC-SSS-GE11 in normal female mice is represented in Table 1. Low accumulation of this radiolabeled peptide was observed in heart, lung, salivary gland, stomach, muscle and bone. Negligible amount of the radioactivity accumulation in stomach, thyroid and salivary gland indicated high in vivo stability of the radiolabeled peptide with low release of free ^99m^Tc. Also the percent of the injected dose of the radiolabeled peptide that accumulated in the liver were 2.88, 2.05 and 0.33% at 1, 4 and 24 h, respectively. In addition, % ID/g values for radiolabeled peptide in the kidneys at 1, 4 and 24 h were 24.5, 17.5 and 3.5%, respectively. These findings showed that the radiolabeled peptide has low tendency for accumulation in liver and excretion by hepatobiliary system and as a result the kidney was the main excretion rout for the radiolabeled peptide. Also, the blood level of radioactivity was less than 1% ID/g at 1 and 4 h because the radiolabeled conjugate was cleared rapidly from the blood.

**Table 1:** Biodistribution of ^99m^Tc-Tricine-HYNIC-SSS-GE11 peptide in normal female mice at 1, 4 and 24 h p.i.

	% ID/g ± SD		
Tissue	1 h	4 h	24 h
Blood	0.84 ± 0.04	0.39 ± 0.11	0.18 ± 0.05
Heart	0.76 ± 0.16	0.44 ± 0.18	0.40 ± 0.2
Lung	1.14 ± 0.63	0.68 ± 0.23	0.47 ± 0.09
s.g & Th.^a^	0.87 ± 0.15	0.46 ± 0.22	0.36 ± 0.12
Liver	2.88 ± 0.84	2.05 ± 0.39	0.33 ± 0.069
Spleen	0.61 ± 0.14	0.41 ± 0.13	0.20 ± 0.06
Kidney	24.5 ± 1.7	17.5 ± 1.25	3.55 ± 0.38
Stomach	0.91 ± 0.27	0.57 ± 0.15	0.32 ± 0.19
Muscle	0.52 ± 0.14	0.23 ± 0.11	0.19 ± 0.05
Bone	0.97 ± 0.14	0.57 ± 0.2	0.28 ± 0.05
Intestine	3.1 ± 0.91	1.89 ± 0.44	0.93 ± 0.28

Each mouse was administered 1 μg of radiolabeled peptide (*n* = 4) and sacrificed at 1, 4 and 24 h after injection. Data are mean ± SD

^a^Salivary gland and Thyroid are abbreviated as s.g and Th. respectively

### Biodistribution in nude mice bearing SKOV3 human ovarian cancer xenografts

To evaluate the uptake of radiolabeled peptide by ovarian tumor, additional biodistribution studies were performed in nude mice bearing SKOV3 xenografts (Table 2). Results compared to the normal mice showed similar patterns of low liver and high kidney uptakes and a fast clearance from the blood. Tumor uptake of radiolabeled conjugate were 2.33 and 1.66% at 1 and 4 h, respectively. The tumor/muscle ratios were 2.4 and 3.4 at 1 and 4 h, respectively. Also, tumor uptake was reduced significantly from 1.66% at 4 h to 0.93% in blocking study whereas 500-fold molar excess of cold peptide was injected 30 min prior injection of the related radioconjugated peptide.

**Table 2:** Biodistribution of ^99m^Tc-Tricine-HYNIC-SSS-GE11 peptide in mice bearing SKOV3 xenograft at 1 and 4 h after injection

	% ID/g ± SD		
Tissue	1 h	4 h	4 h-blocked^a^
Blood	2.53 ± 0.51	1.43 ± 0.18	0.99 ± 0.18
Heart	2.40 ± 0.96	1.56 ± 0.37	0.65 ± 0.1
Lung	3.39 ± 0.64	1.79 ± 0.34	1.24 ± 0.12
s.g & Th.^b^	2.52 ± 0.82	1.80 ± 0.21	0.89 ± 0.16
Liver	6.93 ± 0.95	4.64 ± 0.69	3.28 ± 0.93
Spleen	2.86 ± 0.17	1.49 ± 0.32	0.83 ± 0.1
Kidney	64.95 ± 7.81	57.90 ± 0.49	42.10 ± 4.39
Stomach	2.65 ± 0.66	1.53 ± 0.16	1.16 ± 0.11
Muscle	0.99 ± 0.21	0.51 ± 0.14	0.51 ± 0.07
Bone	1.91 ± 0.53	1.29 ± 0.29	1.01 ± 0.11
Intestine	8.38 ± 4.11	3.24 ± 0.9	3.83 ± 2.1
Tumor	2.33 ± 0.67	1.66 ± 0.41	0.93 ± 0.10
Tumor/Muscle Ratio	2.3	3.42	1.86
Tumor/Bone Ratio	1.2	1.3	0.92
Tumor/Blood Ratio	0.92	1.17	0.96

^a^To block receptors, mice were pretreated with excess of unlabeled peptides (500 μg) by intraperitoneal injection at 0.5 h before radiolabeled peptide injection (*n* = 3)

Nude mice were administered 1 μg of radiolabeled peptide (*n* = 4) and sacrificed at 1 and 4 h after injection. Data are mean ± SD

^b^Salivary gland and Thyroid are abbreviated as s.g and Th. respectively

## Discussion

Radionuclide diagnostic strategy through utilization of molecular targeting probe is a simple and non-invasive method for diagnosis as well as treatment of cancers. EGFR has been reported to have a crucial role in initiation, progression and invasion of a variety of human malignancies with epithelial origin [28]. GE11 is a small peptide which was introduced by Li et al. during investigations on phage display techniques [11]. It has been shown that GE11 is able to bind EGFR efficiently with a K_D_ of 22nM without mitogencity. In this study GE11 was labeled with ^99m^Tc to introduce a potential radiolabelled peptide for in vivo targeting of tumors having overexpression of EGFR. Results of this study showed that radiolabeled GE11 peptide displays moderate tumor uptake, rapid blood clearance and high renal and low hepatobiliary excretion. Several chelators such as diaminedithiol (N_2_S_2_), triamidethiol (N_3_S), PnAO (N_4_) and HYNC have been used for labelling of a peptide with ^99m^Tc. In this study HYNIC was used as chelator due to simple and high yield labeling and appropriate biological properties [29]. Tricine was selected as a coligand because it provides acceptable radiolabeling efficacy [22]. To improve pharmacokinetic properties and renal excretion of the labeled peptide, SSS linker was inserted between chelator and N-terminus of GE11 peptide [30]. This approach resulted in generation of a radiolabeled peptide with high radiochemical yield (>98%) and acceptable stability in solution as it was verified by ITLC. Instability of ^99m^Tc-tricine-complex due to multiple coordination isomerism have been described previously [31]. Assessment of the stability of the radiolabeled peptide of this investigation in human serum showed promising results. The radiochemical purity was 95% within 4 h incubation time and no significant impurities including ^99m^TcO_4_^−^, RHT and ^99m^Tc-coligand were released.

It has been reported previously that GE11 is able to bind EGFR especially those of human hepatoma cell line SMMC-7721 [11], human non-small cell lung carcinoma cell line H1299 [12], human glioblastoma astrocytoma U87-MG [32], non-small cell lung cancer A549 [33], human ovarian adenocarcinoma SKOV3 [14, 16] and breast cancer HCC70 cells [34]. In this study the tendency of ^99m^Tc-tricine-GE11 complex in binding to SKOV3, A549 and MCF-7 cell lines were compared. These cell lines have different levels of HER1 and HER2 expression. Several studies have shown that both epithermal growth factor 1 and 2 are associated and expressed at high levels in human ovarian cancer cell line SKOV3 and human non-small cell line cancer A549 but have lower expression of HER1 and HER2, respectively [17, 35, 36]. HER2 has important role in proliferation and progression of a variety of cancers such as breast, ovarian, non-small cell lung and hepatoma [37]. Therefore, several investigations have utilized different types of targeting biomolecules such as antibody, affibody, aptamer and peptide for diagnose and follow up of the treatments of cancers with high level of HER2 expression [38–45]. These targeting biomolecules have some disadvantages such as high molecular weight, high cost, long-half life in blood circulation, high immunogenicity and low stability. Recent studies have shown that the use of GE11 as an EGFR targeting biomolecule enhances the delivery of chemotherapeutic agents to ovarian cancer cells [14, 46].

In this work by competition and blocking experiments and using FDA approved antibodies it was shown that GE11 radioconjugate has high tendency to bind to HER2 in SKOV3 cell line. Saturation binding experiments showed that the ^99m^Tc-tricine-HYNIC-SSS-GE11 complex was able to bind to SKOV3 with a K_D_ of 73 nM. The high in vivo stability was shown by biodistribution experiments in normal female mice where there was no significant release of the free ^99m^Tc in salivary glands and stomach. The % ID/g values of the blood uptake of ^99m^Tc-tricine-HYNIC-SSS-GE11 complex were 0.83, 0.39 and 0.18 at 1, 4 and 24 h respectively, indicating rapid clearance of the radioconjugate from the blood. In general, the high activity in gastrointestinal is very problematic and may mask metastatic lesions of other target organs. Several reports have shown the importance of linkers in modification of pharmacokinetic by reducing non-target uptake and improving tumor visualization [20, 21, 47]. In contrast to results of the first report on biodistribution of GE11 labeled with ^125^I which showed high liver uptake [11], the kidney was the main route of excretion of radiolabeled GE11 peptide of this study due to the presence of seryl-seryl-serine as pharmacokinetic modifier linker. Results of in vivo biodistribution in nude mice bearing SKOV3 cancer xenografts, showed that the radioconjugated peptide had moderate tumor uptake with % ID/g values of 2.33 and 1.66 at 1 and 4 h after injection respectively. There was a significant statistical differences between concentration of the radiolabeled peptide in tumor and muscle in the way that the tumor-to-muscle ratio at 1 and 4 h of post-injection were 2.3 and 3.42, while tumor-to-blood ratio at these periods of time were 0.92 and 1.17 respectively. Results of this investigation shows that further optimization might be required to increase the clearance rate of the prepared radioconjugated peptide without reducing concentration of tumor radioactivity.

## Conclusions

In this study the ^99m^Tc-tricine-HYNIC-SSS-GE11 was prepared with highly radiochemical purity and good stability. The radioconjugated peptide showed specific binding mediated by HER2 on the cell surface. The biodistribution of the prepared radioconjugated peptide in normal and SKOV3 xenograft mice model showed that the renal system is the main route for excretion of the radioactivity due to presence of SSS linker which was used for modification of the pharmacokinetic. A tumor-to-muscle ratio of 3.4 was obtained with ^99m^Tc-labeled peptide that showed HER2 targeting on SKOV3 tumor.

## Abbreviations

^99m^Tc: 99 m-Technetium
DMEM: Dulbecco’s Modified Eagle’s Medium
EGFR: Epidermal Growth Factor Receptor
FBS: Fetal Bovine Serum
HER: Human Epidermal growth factor Receptor
HYNIC: Hydrazinonicotinic acid
ITLC-SG: Instant Thin Layer Chromatography on Silica Gel
MAbs: Monoclonal Antibodies
MEK: Methyl Ethyl Ketone
NSCLC: Non-Small Cell Lung Cancer
RHT: Reduced Hydrolyzed Technetium
RP-HPLC: Reversed-Phase High-Performance Liquid Chromatography
SCCHN: Squamous Cell Carcinoma of the Head and Neck
SSS-GE11: Seryl-Seryl-Seryl-GE11 peptide
TFA: Trifluoroacetic acid
